# Prognostic value of a systemic inflammatory response index in metastatic renal cell carcinoma and construction of a predictive model

**DOI:** 10.18632/oncotarget.10626

**Published:** 2016-07-16

**Authors:** Liangyou Gu, Xin Ma, Lei Wang, Hongzhao Li, Luyao Chen, Xintao Li, Yu Zhang, Yongpeng Xie, Xu Zhang

**Affiliations:** ^1^ Department of Urology/State Key Laboratory of Kidney Diseases, Chinese PLA General Hospital/PLA Medical School, Beijing, P.R. China; ^2^ School of Medicine, Nankai University, Tianjin, China

**Keywords:** inflammation, metastatic renal cell carcinoma, prognosis, biomarker, nomogram

## Abstract

Inflammation act as a crucial role in carcinogenesis and tumor progression. In this study, we aim to investigate the prognostic significance of systemic inflammatory biomarkers in metastatic renal cell carcinoma (mRCC) and develop a survival predictive model. One hundred and sixty-one mRCC patients who had undergone cytoreductive nephrectomy were enrolled from January 2006 to December 2013. We created a systemic inflammation response index (SIRI) basing on pretreatment hemoglobin and lymphocyte to monocyte ratio (LMR), and evaluated its associations with overall survival (OS) and clinicopathological features. Pretreatment hemoglobin and LMR both remained as independent factors adjusted for other markers of systemic inflammation responses and conventional clinicopathological parameters. A high SIRI seems to be an independent prognosis predictor of worse OS and was significantly correlated with aggressive tumor behaviors. Inclusion of the SIRI into a prognostic model including Fuhrman grade, histology, tumor necrosis and targeted therapy established a nomogram, which accurately predicted 1-year survival for mRCC patients. The SIRI seems to be a prognostic biomarker in mRCC patients. The proposed nomogram can be applied to predict OS of patients with mRCC after nephrectomy.

## INTRODUCTION

Renal cell carcinoma (RCC) accounts for 2–3% of all malignant diseases in adults [1, 2]. Approximately 20% to 30% of patients accompany metastatic disease at the initial diagnosis. Moreover, 30% of patients experience progress to metastatic or locally recurrent disease after nephrectomy for localized disease [3]. Over the past decade, the treatment strategy for metastatic renal cell carcinoma (mRCC) has remarkably developed. Due to the advent of targeted therapy, the outcome of patients with mRCC has been improved [4]. This has been achieved primarily through the elucidation of the considerable role of vascular endothelial growth factor (VEGF) and mammalian target of rapamycin (mTOR) pathways in RCC. However, a better understanding of the pathogenesis of this tumor is still greatly needed [5]. By far, clinical trials and retrospective multivariate analyses has found several clinical prognostic markers, which result in the establishment of prognostic models [6–8]. Nevertheless, accurate prediction of individual tumor biology is still hard. According to anticipation, combining specific RCC biomarkers with routine clinicopathological parameters can realize better prediction of oncologic outcomes [9].

Increasing evidence suggests that inflammatory cells are an essential component of the tumor microenvironment, the inflammatory response serve as a crucial role in cancer development and progression and may be associated with systemic inflammation [10, 11]. The systemic inflammatory response, which is usually evaluated based on surrogate peripheral blood-based parameters, such as C-reactive protein, neutrophil, or platelet count, has been reported to independently associated with oncologic outcomes in various cancers [12, 13]. Several of these parameters have been converted to ratios, such as the neutrophil to lymphocyte ratio (NLR) [14, 15], platelet to lymphocyte ratio (PLR) [16, 17] and lymphocyte to monocyte ratio (LMR) [18, 19], which have been broadly found to be important prognosis predictors. Preoperative hemoglobin and serum albumin levels are also identified as predictors for oncologic outcomes [20, 21]. As independent indicators, we analyzed them all together and then try to apply them to optimize prognosis prediction for mRCC patients.

In this study, we aimed to evaluate the prognostic significance of systemic inflammatory biomarkers in mRCC. We combined preoperative hemoglobin and LMR to develop a novel prognostic marker, named systemic inflammatory response index (SIRI). The relationships of SIRI with clinicopathologcal parameters and overall survival were investigated. Finally, a nomogram basing on SIRI and other independent prognosis predictors was constructed to predict 1-year and 2-year survival for mRCC patients after cytoreductive nephrectomy.

## RESULTS

### Associations of hemoglobin, LMR and SIRI with OS

The clinicopathological characteristics of included patients are shown in Table 1. Associations of variables and overall survival (OS) were firstly assessed by univariate analysis. The results indicated T stage, Fuhrman grade, histology, tumor necrosis, targeted therapy as well as hemoglobin, serum albumin, NLR, PLR and LMR as continuous variables were prognostic factors for OS, whereas other variables didn't obtain statistical difference (Table 2). The significant parameters in univariate analysis were then included to assess associations with OS by multivariate analysis. The results identified that hemoglobin (HR, 0.982; 95% CI, 0.973-0.991; *P* < 0.001) and LMR (HR, 0.844; 95% CI, 0.735-0.969; *P* = 0.016) can independently predict OS, together with Fuhrman grade, tumor necrosis and the absence of targeted therapy (Table 2).

**Table 1:** Baseline patient characteristics

Characteristics	No. (%)
Age (years), median (min-max)	56 (17–83)
Gender	
Male	128 (80%)
Female	33 (20%)
Presentation	
Incidental	86 (53%)
Symptomatic	75 (47%)
Nephrectomy	
Minimally invasive	76 (47%)
Open	85 (53%)
Tumor site	
Left	80 (50%)
Right	81 (50%)
Tumor size (cm)	
≤ 7	84 (52%)
> 7	77 (48%)
T stage	
T1	62 (39%)
T2	28 (16%)
T3	62 (39%)
T4	9 (6%)
N stage	
N0	125 (78%)
N1	36 (22%)
Fuhrman grade	
G1 + G2	73 (46%)
G3 + G4	85 (54%)
Histology	
Clear cell	145 (90%)
Non-clear cell	16 (10%)
Tumor necrosis	
Absent	89 (55%)
Present	72 (45%)
Microvascular invasion	
Absent	107 (66%)
Present	54 (34%)
Metastatic sites	
Bone	48 (30%)
Liver	30 (19%)
Lung	82 (51%)
Other	26 (16%)
Number of metastatic site	
< 2	139 (86%)
≥ 2	22 (14%)
Targeted therapy	
Absent	62 (39%)
Present	99 (61%)

No. = number of patients.

**Table 2:** Univariate and multivariate analysis of prognostic factors of overall survival by Cox regression model

Parameters	Univariate			Multivariate^a^			Multivariate^b^		
	HR	95% CI	*P*–value	HR	95% CI	*P*–value	HR	95% CI	*P*–value
Age at diagnosis (years)			0.796						
≤ 60	1 (Ref)								
> 60	1.064	0.666–1.699							
Gender	0.896	0.607–1.322	0.580						
Presentation			0.112						
Incidental	1 (Ref)								
Symptomatic	1.353	0.931–1.966							
Nephrectomy			0.066						
Minimally invasive	1 (Ref)								
Open	1.429	0.977–2.089							
Tumor site	0.842	0.580–1.224	0.368						
Tumor size (cm)			0.188						
≤ 7	1 (Ref)								
> 7	1.288	0.884–1.876							
T stage			0.005			0.775			0.835
T1 + T2	1 (Ref)			1 (Ref)			1 (Ref)		
T3 + T4	1.704	1.173–2.476		1.071	0.696–1.648		1.049	0.671–1.638	
N stage			0.235						
N0	1 (Ref)								
N1	1.301	0.843–2.010							
Fuhrman grade			< 0.001			0.024			0.010
G1 + G2	1 (Ref)			1 (Ref)			1 (Ref)		
G3 + G4	2.236	1.519–3.291		1.635	1.068–2.504		1.728	1.142–2.616	
Histology			0.002			0.086			0.028
Clear cell	1 (Ref)			1 (Ref)			1 (Ref)		
Non–clear cell	2.579	1.432–4.645		1.695	0.928–3.096		1.966	1.076–3.594	
Tumor necrosis			0.008			0.006			0.001
Absent	1(Ref)			1 (Ref)			1 (Ref)		
Present	1.673	1.147–2.440		1.774	1.175–2.678		1.976	1.325–2.946	
Microvascular invasion			0.155						
Absent	1(Ref)								
Present	1.324	0.900–1.947							
Number of metastatic site			0.085						
< 2	1(Ref)								
≥ 2	1.568	0.939–2.617							
Targeted therapy			< 0.001			< 0.001			< 0.001
Absent	1(Ref)			1 (Ref)			1 (Ref)		
Present	0.360	0.247–0.524		0.273	0.183–0.409		0.324	0.216–0.487	
Hemoglobin c	0.979	0.971–0.987	< 0.001	0.982	0.973–0.991	< 0.001			
Albumin c	0.952	0.916–0.988	0.010	1.027	0.975–1.081	0.317			
NLR c	1.125	1.067–1.187	< 0.001	0.896	0.725–1.107	0.310			
PLR c	1.004	1.002–1.005	< 0.001	1.001	0.999–1.003	0.147			
LMR c	0.809	0.709–0.923	0.002	0.844	0.735–0.969	0.016			
SIRI			< 0.001						< 0.001
0							1 (Ref)		
1							1.785	1.091–2.919	0.021
2							2.732	1.639–4.556	< 0.001

HR = hazard ratio; CI = confidence interval; Ref = Referent; NLR = neutrophil to lymphocyte ratio; PLR = platelet to lymphocyte ratio; LMR = lymphocyte to monocyte ratio; SIRI = systemic inflammation response index.

^a^Adjustment for T stage, Fuhrman grade, histology, tumor necrosis, targeted therapy, hemoglobin, serum albumin, NLR, PLR and LMR.

^b^Adjustment for T stage, Fuhrman grade, histology, tumor necrosis, targeted therapy and SIRI.

^c^Analyzed as a continuous variable.

**Figure 1:**
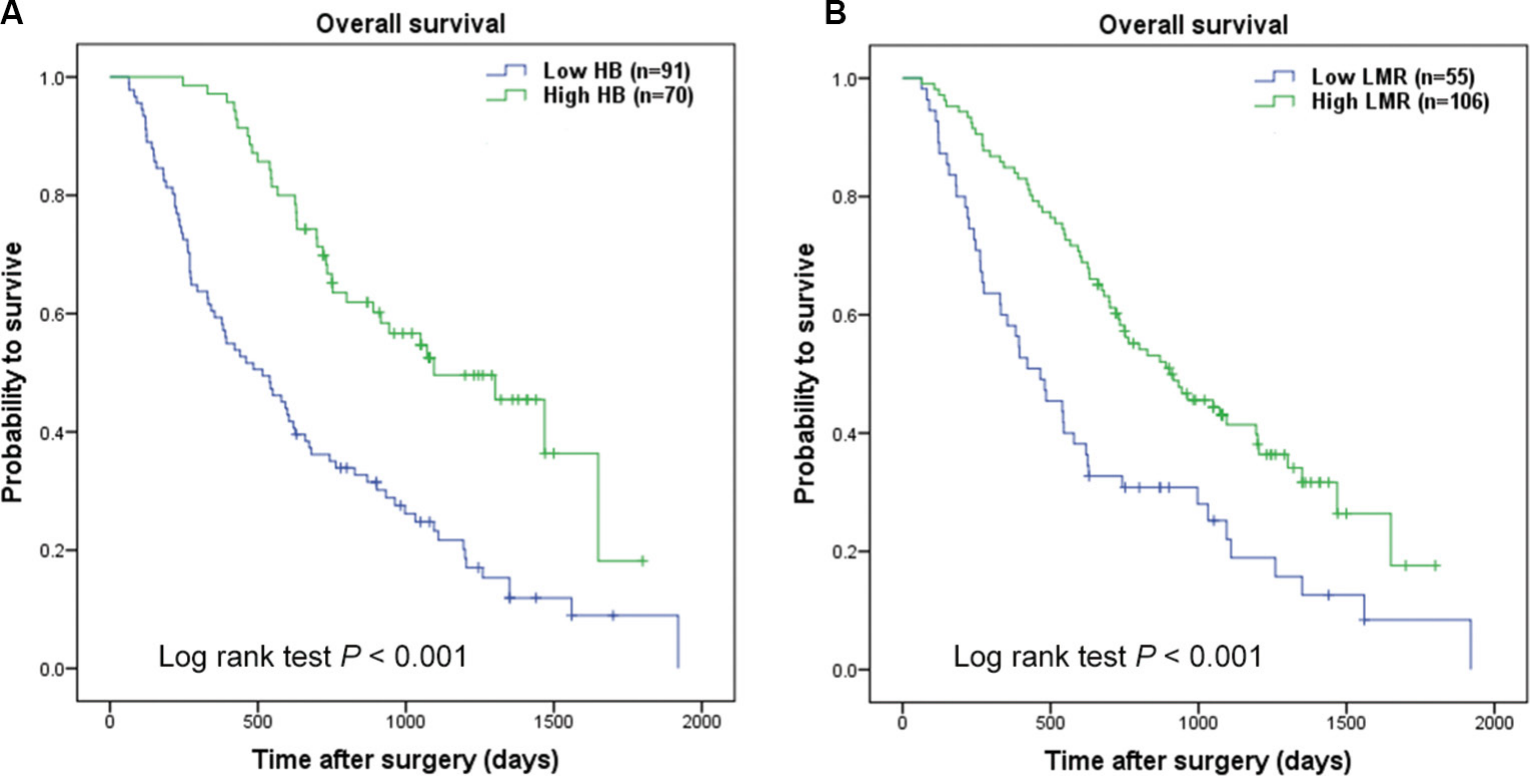
Kaplan-Meier curves for overall survival probability according to preoperative hemoglobin and LMR Kaplan-Meier analysis for OS according to (**A**) preoperative hemoglobin, (**B**) preoperative LMR.

**Figure 2:**
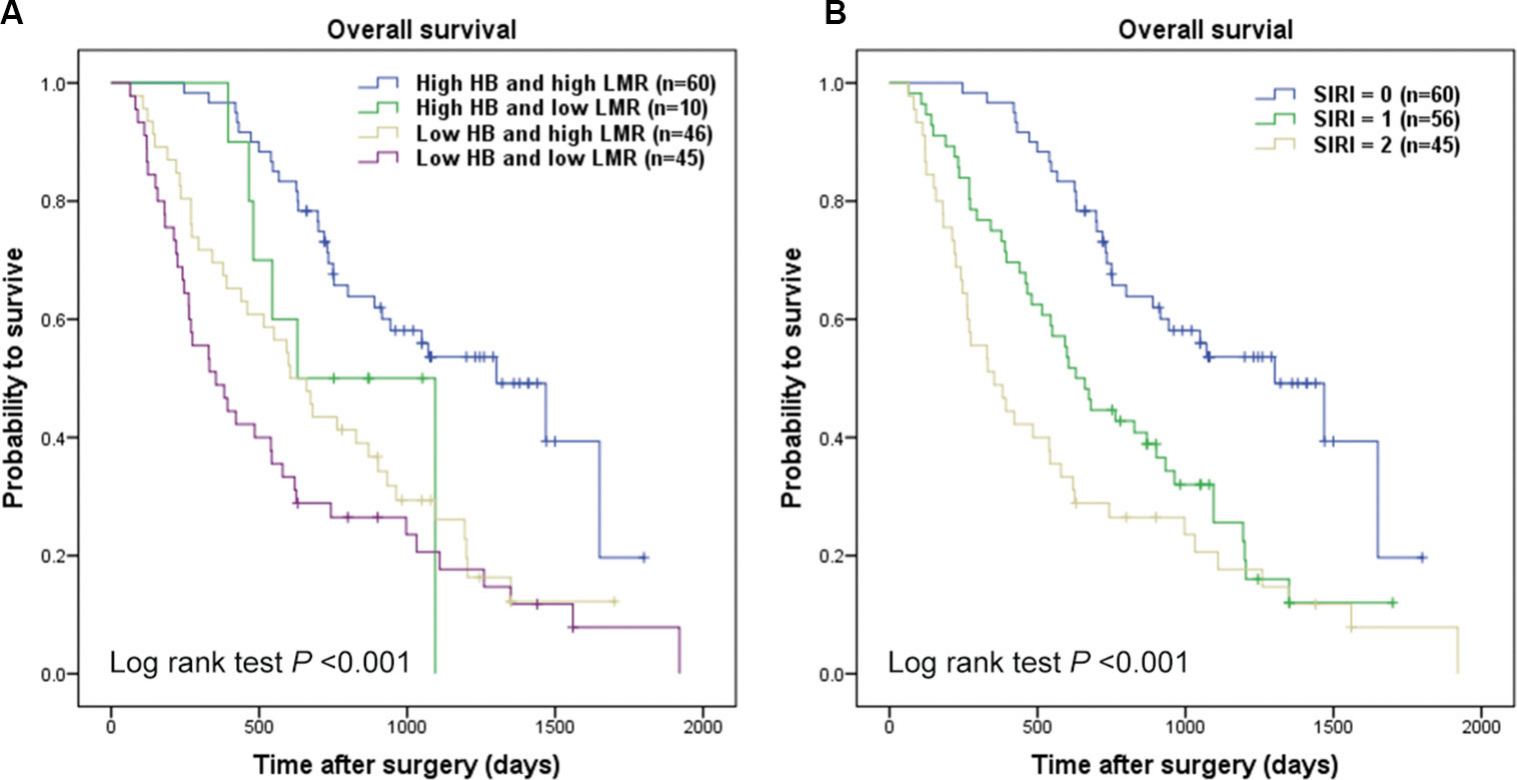
Kaplan-Meier curves for overall survival probability according to combination of preoperative hemoglobin and LMR Kaplan-Meier analysis for OS according to (**A**) combination of preoperative hemoglobin and LMR, (**B**) SIRI.

As mentioned in the methods section, cut-point of hemoglobin was 137/116 gl^−1^ (137 gl^−1^ for male and 116 gl^−1^ for female), the optimal cut-off level for LMR was 3.23. The ROC curve was seen in Supporting Data Figure S1. Kaplan-Meier survival analysis indicated that hemoglobin (< 137/116 gl^−1^) and LMR (< 3.23) were both significantly correlated with decreased OS (*P* < 0.001 for both) (Figure 1). Hemoglobin and LMR as categorical variables also were independent prognosis predictors in multivariate analysis (*P* < 0.001 for both). To further distinguish patients with different clinical prognosis, we combined hemoglobin with LMR value to set four subgroups. And significant differences were found among the four subgroups (*P* < 0.001; Figure 2A). Since there were no statistical difference in subgroups of high hemoglobin and low LMR or low hemoglobin and high LMR (log-rank *P* = 0.526), and deficient subjects in high hemoglobin and low LMR subgroup, we merged the two subgroups. The SIRI was defined as following: patients with both elevated hemoglobin and elevated LMR (≥ 137/116 gl^−1^ and ≥ 3.23, respectively) were allotted to group 0; patients with either elevated hemoglobin or elevated LMR were allotted to group 1; patients with both decreased hemoglobin and decreased LMR (< 137/116 gl^−1^ and < 3.23, respectively) were assigned to group 2. Kaplan-Meier analysis identified that a high SIRI was significantly correlated with reduced OS (*P* < 0.001; Figure 2B).

The univariate analysis revealed that the SIRI has prognostic significance for OS (*P* < 0.001). In the multivariate analysis, the SIRI was independent prognostic predictor for OS. Taking group 0 as a reference, the HR for group 1 was 1.785 (95% CI, 1.091-2.919; *P* = 0.021), the HR for group 2 was 2.732 (95% CI, 1.639-4.556; *P* < 0.001). Also, Fuhrman grade (*P* = 0.01), histology (*P* = 0.028), tumor necrosis (*P* = 0.001) and targeted therapy (*P* < 0.001) were independent prognosis predictors of OS in mRCC patients (Table 2).

### Correlations of hemoglobin, LMR and SIRI with clinicopathological parameters

Comparisons analyses indicated that decreased hemoglobin and LMR were both significantly correlated with the presence of symptom (*P* = 0.015 and *P* = 0.002, respectively), higher T stage (*P* < 0.001 for both), higher Fuhrman grade (*P* < 0.001 for both), the presence of microvascular invasion (*P* < 0.001 and *P* = 0.003, respectively). Additionally, decreased LMR was associated with the presence of tumor necrosis (*P* = 0.032) (Table 3).

**Table 3:** Associations of Hemoglobin, LMR and SIRI with clinicopathological parameters

Parameters	Hemoglobin (g/dl)			LMR			SIRI			
	< 13.7/11.6	≥ 13.7/11.6	*P*-value	< 3.23	≥ 3.23	*P*-value	0	1	2	*P*-value
	*n* = 91	*n* = 70		*n* = 55	*n* = 106		*n* = 60	*n* = 56	*n* = 45	
Age (years)			0.493			0.391				0.991
≤ 60	55	46		37	64		40	30	31	
> 60	36	24		18	42		20	26	14	
Gender			0.797			0.600				0.648
Male	73	55		45	83		47	44	37	
Female	18	15		10	23		13	12	8	
Presentation			0.015			0.002				0.001
Incidental	41	45		20	66		40	31	15	
Symptomatic	50	25		35	40		20	25	30	
Tumor site			0.376			0.824				0.499
Left	48	32		28	52		28	28	24	
Right	43	38		27	54		32	28	21	
Tumor size (cm)			0.081			0.004				0.006
≤ 7	42	42		20	64		38	30	16	
> 7	49	28		35	42		22	28	29	
T stage			< 0.001			< 0.001				< 0.001
T1 + T2	38	52		20	70		45	32	13	
T3 + T4	53	18		35	36		15	24	32	
N stage			0.312			0.497				0.305
N0	68	57		41	84		49	43	33	
N1	23	13		14	22		11	13	12	
Fuhrman grade			< 0.001			< 0.001				< 0.001
G1 + G2	28	45		16	57		38	26	9	
G3 + G4	61	24		39	46		21	28	36	
Histology			0.298			0.394				0.251
Clear Cell	80	65		48	97		56	50	39	
Non-clear Cell	11	5		7	9		4	6	6	
Tumor necrosis			0.169			0.032				0.039
Absent	46	43		24	65		37	34	18	
Present	45	27		31	41		23	22	27	
Microvascular invasion			< 0.001			0.003				< 0.001
Absent	48	59		28	79		51	36	20	
Present	43	11		27	27		9	20	25	
Number of metastatic site			0.841			0.229				0.505
< 2	79	60		45	94		55	44	40	
≥ 2	12	10		10	12		5	12	5	

LMR = lymphocyte to monocyte ratio; SIRI = systemic inflammation response index.

The associations between the SIRI and clinicopathologic parameters were also presented in Table 3. Patients in higher SIRI group were more likely to have the presence of symptom (*P* = 0.001), larger tumor size (*P* = 0.006), higher T stage (*P* < 0.001), higher Fuhrman grade (*P* < 0.001), the presence of tumor necrosis (*P* = 0.039) and the presence of microvascular invasion (*P* < 0.001).

### Prognostic nomogram for OS

**Figure 3:**
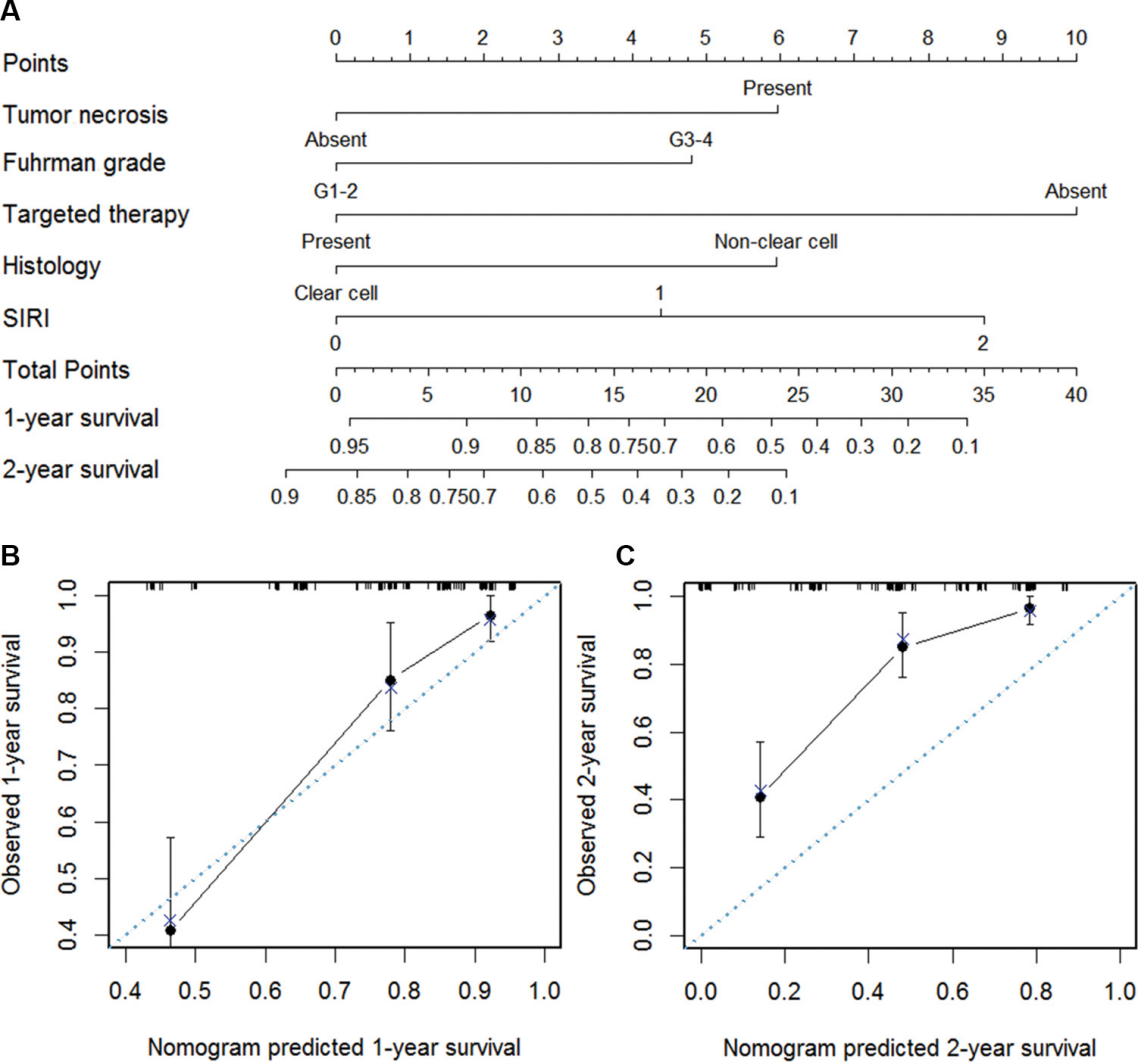
Nomogram for predicting 1- and 2-year OS of mRCC patients after nephrectomy (**A**) Nomogram for predicting 1- and 2-year OS of mRCC patients after nephrectomy. Calibration plot of the nomogram for (**B**) 1-year and (**C**) 2-year survival. The blue dashed line represents the “ideal” line of a perfect match between predicted and observed survival. The black line indicates the performance of the proposed nomogram. Black dots are sub-cohorts of the data set; X is the bootstrapped corrected estimate of nomogram with 300 resamples. Vertical bars represent 95% confidence interval.

To quantitatively predict the survival of mRCC patients after cytoreductive nephrectomy, a prognostic nomogram was generated using all the significant independent indicators including Fuhrman grade, histology, tumor necrosis, targeted therapy and SIRI (Figure 3A). The nomogram can predict the survival probability for mRCC patients within 1 or 2 years after cytoreductive nephrectomy. In the nomogram, a higher total points indicates an inferior outcome, and calibration plots of the nomogram predicting 1-year survival worked well with the constructed model (Figure 3B). As shown in Figure 3C, the trend of observed 2-year survival was higher than the predicted 2-year survival, which means the nomogram have a trend to underestimate 2-year survival in mRCC patients. The C-index of the multivariate prognostic model based on Fuhrman grade, histology, tumor necrosis, targeted therapy was 0.72 and enhanced to 0.75 by the inclusion of SIRI (*P* = 0.007).

## DISCUSSION

In the present study, we studied clinicopathological features and prognosis of 161 mRCC patients. We confirmed that hemoglobin and LMR were independent prognostic factors and adversely predicted OS of mRCC patients. Though serum albumin, NLR and PLR were significant indicators in univariate analysis, they were not independently associated with survival in the multivariate model. Moreover, we created a new prognostic marker named SIRI based on dichotomous hemoglobin and LMR. We found that high SIRI was associated with poor outcome and large tumor size, high T stage, high Fuhrman grade and the presence of tumor necrosis and microvascular invasion. Hence, SIRI could be a more objective and relatively available marker to improve the predictive accuracy. This study tries to form a nomogram to predict the survival probability of mRCC patients after cytoreductive nephrectomy within 1-year and 2-year based on Fuhrman grade, histology, tumor necrosis, targeted therapy and SIRI. The C-index for the nomagram is 0.75. Calibration plots of the nomogram predicting 1-year survival worked well with the constructed model. However, the nomogram have a trend to underestimate 2-year survival in mRCC patients, which need further optimization.

Recently, several inflammatory biomarkers have been identified in RCC. In non-metastatic RCC, the prognostic significance of NLR and LMR in patients after surgery were reported [14, 22]. In metastatic RCC, the value of NLR also has been proven [5, 23, 24]. And the prognostic role of PLR was indicated in patients with advanced RCC [16]. Moreover, Karakiewicz et al. [25] revealed that pretreatment high hemoglobin was significantly correlated with superior cancer-specific survival for 1828 all-stages RCC patients. Another study in 369 locoregional RCC patients identified that pretreatment serum albumin can significantly predict oncologic outcomes [26]. Nevertheless, the prognostic significance of combining these frequently reported hematological and laboratory markers remains obscure in mRCC.

As a merged biomarker based on hemoglobin and LMR, the biological reason why SIRI could be of prognostic relevance could be explained by the function of hemoglobin, lymphocytes and monocytes. Several mechanisms whereby malignancy induces low hemoglobin have been suggested, including blood loss, functional iron deficiency, and inflammation leading to reductions in renal erythropoietin production [27]. Recent evidence indicates that anemia could be an important contributor to a more aggressive cancer biology and worse prognosis, presumably by affecting tumor hypoxia and decreasing quality of life and treatment delivery [28]. Anemia may also be a presentation of patient's physical weakness, under-nutrition, and susceptibility of infection; hence, a preoperative predisposition to poor general health condition may lead to poor outcome.

The LMR could be an excellent reflection of cancer, lymphopenia is a surrogate marker of weak immune response, high level of monocyte count reflect a high tumor burden. Lymphocytes significantly mediate the process of immunosurveillance and immune-editing, and their lymphocyte infiltration into the tumor microenviroment is a requirement to an immunologic anti-tumor reaction [29, 30]. In general, a low lymphocyte count could partly explain the weak, deficient immunologic reaction to the tumor [29]. Nevertheless, monocytes infiltrating tumor tissue also have an effect on tumor development and progression [10], which exert a major role in innate immunity [31]. Recent evidence indicates that monocytes can differentiate into tumor-associated macrophages (TAMs) enhancing tumor progression [32]. Pollard and Condeelis et al. [33, 34] found that macrophages support tumor cell migration, invasion and intravasation as well as tumor-associated angiogenesis and even result in a suppression of anti-tumor immune reaction. Moreover, Lin et al. [35] and Jetten et al. [36] gave insight into the role of macrophages in angiogenesis and vascular remodeling induced by them in tumor formations. All this data suggests a pro tumorous potency of monocytes because of formation of diverse macrophage phenotypes that facilitate the malignant process.

The evaluation of SIRI relies on routine laboratory tests of hemoglobin, lymphocyte and monocytes counts, which are relatively easy to obtain in the clinical practice. The advantage of SIRI can facilitate its use in clinical decision-making. Nevertheless, some limitations of this study needed to be acknowledged. Firstly, the study was retrospectively designed, with a small population size of 161 patients. Moreover, because of deficit patients, there were no external validation for the proposed nomogram, which will be verified in the following patient cohort. Secondly, because of incomplete database in our institution, we can't obtain detailed information about some variables in well-established models (IMDC and MSKCC) for part of patients in our cohort. Hence, it is difficult for us to compare our model with the two well-established models. Third, there was some difference in the treatment strategy for patients after cytoreductive nephrectomy, which result in various oncologic outcomes.

In general, we created a new and easily assessed prognostic marker named SIRI, which relied on pretreatment hemoglobin and LMR. The SIRI seems to be an independent prognosis predictor and should be combined with conventional clinicopathological parameters to improve outcome prediction of mRCC patients after nephrectomy.

## MATERIALS AND METHODS

### Patients

This retrospective study examined the records of a sequential series of 161 patients with a new diagnosis of mRCC between January 2006 and December 2013 in our center. The inclusion criteria were as following: 1) All patients with mRCC underwent a cytoreductive nephrectomy; 2) Unilateral renal cancer; 3) No hematology disease, infection, hyperpyrexia; 4) Preoperative blood parameter data available. Informed consent was obtained from all patients and the study was approved by Medical Ethics Committee of our hospital.

The following clinical and pathologic variables were collected: age at surgery; gender; presentation; nephrectomy pattern; primary cancer characteristics (tumor site, tumor size, T stage, N stage, Fuhrman grade, histology, tumor necrosis, microvascular invasion); metastatic sites and number; targeted therapy. The presentation mode was categorized as symptomatic or incidental. Tumors accompanied by hematuria, pain, abdominal mass, fever or weight loss were categorized as symptomatic tumors. Nephrectomy pattern was categorized as minimally invasive or open. Robotic and laparoscopic nephrectomy were categorized as minimally invasive surgery. Primary lesions were staged based on the 2011 UICC TNM classification and graded according to the Fuhrman grading system [37]. Histology was classified to clear cell and non-clear cell. Microvascular invasion refers to the presence of tumor within microscopic or veins with a muscular coat or the lymphatic system, or both. Synchronous lesions were considered as metastases diagnosed at the moment of primary nephrectomy. Targeted therapy included Sorafenib and Sunitinib. The hematological and laboratory data were collected from a time frame of < 1 week prior to nephrectomy and used to calculate NLR, PLR and LMR.

After operation, each patient was followed up regularly until June 2015. Physical examination, laboratory tests, chest imaging and abdominal ultrasound or computed tomography were conducted at every visit. Overall survival (OS) was calculated from operation to death from all causes.

### Statistical analysis

All continuous data were tested for normality. Chi-square test or Fisher's exact test was applied to compare dichotomized variables, and Wilcoxon rank-sum test or Kruskal-Wallis test was applied to compare other categorical variables between groups. Survival curves were compared by Kaplan-Meier survival analysis and was tested by Log-rank test. Univariable and multivariable survival analyses were performed using Cox proportional hazards models. These hematological and laboratory markers including hemoglobin, serum albumin, NLR, PLR and LMR were first evaluated as continuous variables, combined with some clinicopathological parameters. And we found that hemoglobin and LMR were independent prognosis predictor for OS. Then the two factors were analyzed as dichotomized variables. Cut-point of hemoglobin referred to the low range of normal measurement at 137/116 gl^−1^ (137 gl^−1^ for male and 116 gl^−1^ for female). The optimal cut-off level for LMR was determined by receiver operating curve (ROC) analysis to differentiate between survival and death (using the R software version 3.2.1). The SIRI was established according to hemoglobin and LMR levels. The SIRI and routine clinicopathological variables were evaluated in the multivariate analysis. Nomogram for OS was generated by R 3.2.1 software (Institute for Statistics and Mathematics, Vienna, Austria), and the predictive accuracy was evaluated by Harrell's concordance index (c-index) [38]. Calibration plots were performed to assess the performance characteristics of the predictive nomogram. All statistical analyses were performed using IBM SPSS 20.0 software (IBM, USA). The statistical significance was defined as *P* less than 0.05.

## SUPPLEMENTARY FIGURE


