# Supplementary material for: Prognostic value of a systemic inflammatory response index in metastatic renal cell carcinoma and construction of a predictive model
Source: Oncotarget. 2016 Jul 16;8(32):52094–103. doi: 10.18632/oncotarget.10626 (PMC5581015; doi:10.18632/oncotarget.10626)
Supplement: Supplementary file 1 [file oncotarget-08-52094-s001.pdf]

Prognostic value of a systemic inflammatory response index in metastatic renal cell carcinoma and construction of a predictive model

Supplementary Materials

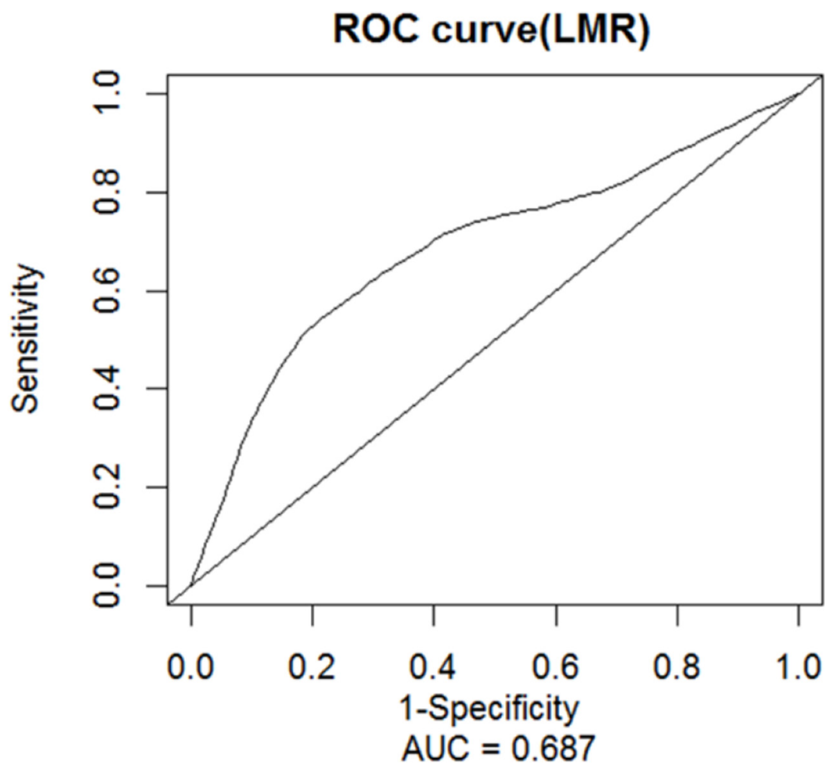

Supplementary Figure S1: Optimal cut-off level for LMR was applied with ROC curves for overall survival.
